# The neuroprotective effects of milk fat globule-EGF factor 8 against oligomeric amyloid β toxicity

**DOI:** 10.1186/1742-2094-9-148

**Published:** 2012-06-28

**Authors:** Endong Li, Mariko Noda, Yukiko Doi, Bijay Parajuli, Jun Kawanokuchi, Yoshifumi Sonobe, Hideyuki Takeuchi, Tetsuya Mizuno, Akio Suzumura

**Affiliations:** 1 Department of Neuroimmunology, Research Institute of Environmental Medicine, Nagoya University, Furo-cho, Chikusa-ku, Nagoya, 464-8601, Japan

**Keywords:** Microglia, Neuroprotection, MFG-E8, Nrf2

## Abstract

**Background:**

Phosphatidylserine receptor is a key molecule that mediates the phagocytosis of apoptotic cells. Milk fat globule-EGF factor 8 (MFG-E8) is a phosphatidylserine receptor that is expressed on various macrophage lineage cells, including microglia in the central nervous system (CNS). Targeted clearance of degenerated neurons by microglia is essential to maintain healthy neural networks. We previously showed that the CX3C chemokine fractalkine is secreted from degenerated neurons and accelerates microglial clearance of neuronal debris via inducing the release of MFG-E8. However, the mechanisms by which microglia produce MFG-E8 and the precise functions of MFG-E8 are unknown.

**Methods:**

The release of MFG-E8 from microglia treated with conditioned medium from neurons exposed to neurotoxic substances, glutamate or oligomeric amyloid β (oAβ) was measured by ELISA. The neuroprotective effects of MFG-E8 and MFG-E8 − induced microglial phagocytosis of oAβ were assessed by immunocytochemistry. The effects of MFG-E8 on the production of the anti-oxidative enzyme hemeoxygenase-1 (HO-1) were determined by ELISA and immunocytochemisty.

**Results:**

MFG-E8 was induced in microglia treated with conditioned medium from neurons that had been exposed to neurotoxicants, glutamate or oAβ. MFG-E8 significantly attenuated oAβ-induced neuronal cell death in a primary neuron − microglia coculture system. Microglial phagocytosis of oAβ was accelerated by MFG-E8 treatment due to increased CD47 expression in the absence of neurotoxic molecule production, such as tumor necrosis factor-α, nitric oxide, and glutamate. MFG-E8 − treated microglia induced nuclear factor E(2) − related factor 2 (Nrf2) − mediated HO-1 production, which also contributed to neuroprotection.

**Conclusions:**

These results suggest that microglia release MFG-E8 in response to signals from degenerated neurons and that MFG-E8 protects oAβ-induced neuronal cell death by promoting microglial phagocytic activity and activating the Nrf2-HO-1 pathway. Thus, MFG-E8 may have novel roles as a neuroprotectant in neurodegenerative conditions.

## Background

Milk fat globule-EGF factor 8 (MFG-E8, lactadherin homolog in humans) is a phosphatidylserine (PS) receptor that acts as a cellular bridge between apoptotic cells and phagocytic cells and triggers the engulfment of cellular debris [1,2]. Recently, we showed that MFG-E8 is expressed in microglia and secreted in response to factors released from degenerated neurons, such as the CX3C chemokine fractalkine (FKN) [3]. FKN-induced MFG-E8 enhances microglial clearance of damaged neuronal debris. MFG-E8 has been shown to improve the pathogenesis of Alzheimer’s disease (AD) by eliminating amyloid β (Aβ) plaques [4]. In addition to microglia, MFG-E8 is also expressed in astrocytes, oligodendrocytes, and neurons [5], although it is uncertain what cell type predominantly produces MFG-E8. MFG-E8 is also implicated in the pathogenesis of immune-related diseases in the periphery. MFG-E8 expression was reduced in lesions associated with murine experimental colitis, and MFG-E8 treatment markedly ameliorated disease progression [6]. This suggests that MFG-E8 acts as a suppressor of the peripheral immune system and that MFG-E8 may be a therapeutic target for immune-mediated bowel diseases [7,8].

Microglia are resident immune cells in the central nervous system (CNS). In neurodegenerative diseases, such as AD and Parkinson’s disease, microglia accumulate in lesions where they are thought to have both neurotoxic and neuroprotective functions [9]. In addition to producing various neuroprotective factors, microglia also phagocytose and remove injured neuronal debris or foreign antigens and this process is critically involved in maintaining healthy neuronal networks. However, the phagocytic activity of microglia is not only beneficial to neurons, but also exerts adverse effects by producing pro-inflammatory factors [10]. In the case of microglial Aβ clearance, various cellular surface receptors are involved in recognizing and appropriately eliminating accumulated Aβ plaques. These include CD14, CD36, CD47, CD200R, PS receptors, Toll-like receptors (TLRs), and receptors for advanced glycation end products (RAGE) [11,12]. Lipopolysaccharide (LPS) activates microglia and increases phagocytosis through the LPS receptor CD14, which may be involved in AD pathology [13,14]. Recent reports indicated that fibrillar Aβ increases CD36 and CD47 expression in microglia [15], and CD36 is required for microglial phagocytosis of Aβ1-42 [16]. CD47 is a key molecule in CNS inflammation that reduces macrophage activation and induces monocyte migration [17]. Intact myelin expresses CD47, which suppresses microglial phagocytosis of myelin [18]. Thus, CD47 seems to act as a “self” marker and inhibits excessive phagocytosis under normal conditions. On the other hand, Aβ phagocytosis correlates with CD47 expression [19], which also suggests that CD47 plays a crucial role in target clearance during pathological conditions.

Aβ-induced oxidative stress is caused by reactive oxygen species (ROS) produced from microglia and astrocytes, and exacerbates the pathological conditions of AD [20,21]. Induction of the nuclear factor E(2) − related factor 2 (Nrf2) signaling pathway could induce neuroprotection against some Aβ-related oxidative stressors.

In the present study, we show that MFG-E8 is released from microglia in response to various soluble factors (for example, FKN) released from degenerated neurons. MFG-E8 increases microglial neuroprotective activity against oAβ-induced neuronal cell death. This neuroprotection is mediated through enhanced microglial phagocytic activity via the CD47 pathway and activation of the Nrf2-HO-1 pathway.

## Materials and methods

### Reagents

L-glutamate and LPS were purchased from Sigma (St. Louis, MO, USA). MFG-E8 was obtained from R&D Systems (Minneapolis, MN, USA). Tin (IV)-mesoporphyrin IX dichloride (SnMP), a specific inhibitor of HO-1, was obtained from Frontier Scientific (Logan, UT, USA), and was dissolved in an arginine-containing solution as previously described [22,23].

### Preparation of oligomeric amyloid β (oAβ) solutions

Aβ1-42 solution was prepared as previously described [24]. Briefly, synthetic human Aβ1-42 (Peptide institute, Osaka, Japan) was dissolved to 1 mM in 100% 1,1,1,3,3,3-hexafluoro-2-propanol (HFIP). HFIP was dried using a vacuum desiccator and resuspended to a concentration of 5 mM in DMSO. To form oligomers, amyloid peptide was diluted to a final concentration of 100 μM with Ham’s F-12, incubated at 4°C for 24 h, and then immediately added to cultures at a final concentration of 5 μM. Formation of oAβ was confirmed using western blot analysis as previously described [24].

### Cell culture

All animal protocols were approved by the Animal Experiment Committee of Nagoya University. Primary neuronal cultures were prepared from the cortices of C57BL/6 mouse embryos at embryonic Day 17 (E17) as previously described [25]. Briefly, cortical fragments were dissociated into single cells in dissociation solution (Sumitomo Bakelite, Akita, Japan), and resuspended in neuron culture medium (Sumitomo Bakelite). Neurons were seeded onto 12-mm polyethyleneimine-coated glass coverslips (Asahi Techno Glass Corp., Chiba, Japan) at a density of 5.0 × 10^4^ cells/well in 24-well plates and incubated at 37°C in a humidified atmosphere containing 5% CO_2_. The purity of the cultures was more than 95% as determined by NeuN-specific immunostaining.

Microglia were isolated from primary mixed glial cell cultures prepared from newborn C57BL/6 mice at Day *in vitro* (DIV) 14 using the “shaking off” method, as previously described [26]. The purity of the cultures was 97 to 100% as determined by immunostaining for the Fc receptor. Cultures were maintained in Dulbecco’s modified Eagle medium supplemented with 10% fetal calf serum, 5 μg/mL bovine insulin and 0.2% glucose. Microglia were seeded at a density of 7.0 × 10^4^ or 1.0 × 10^5^ cells/well in 96- or 48-well plates, respectively.

Neuron–microglia co-cultures were prepared by adding 1.0 × 10^5^ microglia in 100 μL neuronal medium to neuronal cultures (5.0 × 10^4^ neuronal cells) on DIV 14 in 24-well plates. The cultures were maintained in neuron culture medium.

### Measurement of MFG-E8 levels

MFG-E8 secreted from mouse primary microglia or cortical neurons was measured using an ELISA (R&D Systems, Minneapolis, MN, USA) according to the manufacturer’s instructions. Neurons and microglia were treated with oAβ (5 μM) or L-glutamate (20 μM) for 24 h at 37°C. In addition, neuronal conditioned medium (Neu CM) was prepared as follows: 5.0 × 10^4^ neuronal cells in neuronal medium were treated with oAβ (5 μM) or L-glutamate (20 μM) for 24 h, and the supernatant was collected. A total of 1.0 × 10^5^ microglia were treated with Neu CM for 24 h, and then MFG-E8 in the supernatant was measured.

### RT-PCR

Total RNA was extracted from microglia and neurons using an RNeasy Mini Kit (Qiagen, Tokyo, Japan). A first-strand cDNA library was obtained using SuperScript II (Invitrogen, Carlsbad, CA, USA) and oligo (dT)_12–18_ (Invitrogen) as the first-strand primer. Negative control reactions were performed using the same system after heat denaturing the reverse transcriptase. Transcripts encoding mouse CD36, CD47, and glyceraldehydes-3-phosphate dehydrogenase (GAPDH) were amplified by RT-PCR using 0.1 μg of first-strand cDNA, Blend Taq polymerase (Toyobo Co., Osaka, Japan), and oligonucleotide primers (Table 1).

**Table 1:** Oligonucleotide primers of CD14, CD36, CD47 and GAPDH

**Gene**	**Sequence (5’ to 3’)**	**Expected size (bp)**
CD14		
Sense	5’-GACCATGGAGCGTGTGCTTG	464
Antisense	5’-GCCACTGCTGCAGTTCTGCGAG	
CD36		
Sense	5’-GCAAAGAAGGAAAGCCTGTG	367
Antisense	5’-ATCACCACTCCAATCCCAAG	
CD47		
Sense	5’-CTGGTGCTCACAGTCATCGT	213
Antisense	5’-GACCAAAGCAAGGACGTAGC	
GAPDH		
Sense	5’-ACTCACGGCAAATTCAACG	817
Antisense	5’-CCCTGTTGCTGTAGCCGTA	

### Immunocytochemistry

Cells were fixed with 4% paraformaldehyde for 15 minutes at room temperature, blocked with 5% serum, and permeabilized with 0.05% Triton X-100. Neurons were stained with mouse polyclonal anti-MAP-2 antibodies (1:1,000; Chemicon, Temecula, CA, USA) and Alexa 488 − conjugated secondary antibodies (1:1,000; Invitrogen). Microglia were stained with Cy5-conjugated rat anti-mouse CD11b monoclonal antibodies (1:300; BD Biosciences, Franklin Lakes, NJ, USA) prior to fixation. Images were analyzed using a deconvolution fluorescence microscope system (BZ-8000; Keyence Corporation, Osaka, Japan).

Surviving neurons were identified based on their cytoskeletons as previously described [25]. Viable neurons stained strongly with an anti-MAP-2 antibody, whereas damaged neurons showed weaker staining. MAP-2 − positive neurons were counted in representative areas in each well. In five independent experiments, more than 200 neurons were evaluated in each well by a scorer who was blind to the experimental conditions. The number of viable neurons in untreated cultures was set to 100%.

### Evaluation of microglial phagocytosis of oAβ

Microglia were seeded at a density of 6.0 × 10^4^ cells/well in a poly-L-lysine − coated eight-well chamber plate. Cells were pre-treated with MFG-E8 (1 to 100 ng/mL) for 1 h, and then oAβ (5 μM) was added to the culture for 6 h. The cells were subsequently fixed in 4% paraformaldehyde. Microglia were stained with a rabbit polyclonal anti-Rab7 antibody (1:200; Cell Signaling Technology, Danvers, MA, USA) and Alexa 568 − conjugated secondary antibody (1:1,000; Invitrogen). oAβ was stained with a mouse polyclonal anti-4 G8 antibody (1:1000; Chemicon,) and Alexa 488 − conjugated secondary antibody (1:1,000; Invitrogen). Immunostained images were observed under confocal laser scanning microscope (LSM 710; ZEISS, Jena, Germany).

### Fluorescence-activated cell sorting (FACS) analysis of cell surface antigen expression

Microglia were seeded at a density of 1.0 × 10^6^ cells/dish in a 6-cm dish. Cells were treated with 100 ng/mL MFG-E8 for 72 h, and then immunostaining was performed using standard procedures. The primary antibodies included anti-CD36 (1:200; BD Biosciences) and anti-CD47 (1:200; BD Biosciences) and the secondary antibodies were conjugated to FITC (1:1,000; BD Biosciences).

### Measurement of HO-1, tumor necrosis factor-α (TNF-α), nitric oxide (NO), and glutamate

Microglia were treated with LPS or MFG-E8 (1 to 100 ng/mL) and oAβ for 48 h. The supernatants were assessed using a TNF-α ELISA (BD Pharmingen, Franklin Lakes, NJ, USA). Cells were lysed in extraction buffer (1% NP-40 in PBS) and assayed for HO-1 with an ELISA (Takara Bio, Mie, Japan). The NO levels were determined using the Griess reaction as previously reported [27]. To measure glutamate levels, a colorimetric assay kit (Yamasa Corporation, Tokyo, Japan) was used as previously described [28].

### Statistical analysis

Statistically significant differences between the experimental groups were determined by one-way ANOVA followed by Dunnett’s or Tukey’s tests for multiple comparisons. Statistical analyses were performed using Prism 4 for Windows (GraphPad Software, San Diego, CA, USA). *P*-values less than 0.05 were considered significant.

## Results

### Microglia secrete MFG-E8 in response to factors released from degenerated neurons

**Figure 1:**
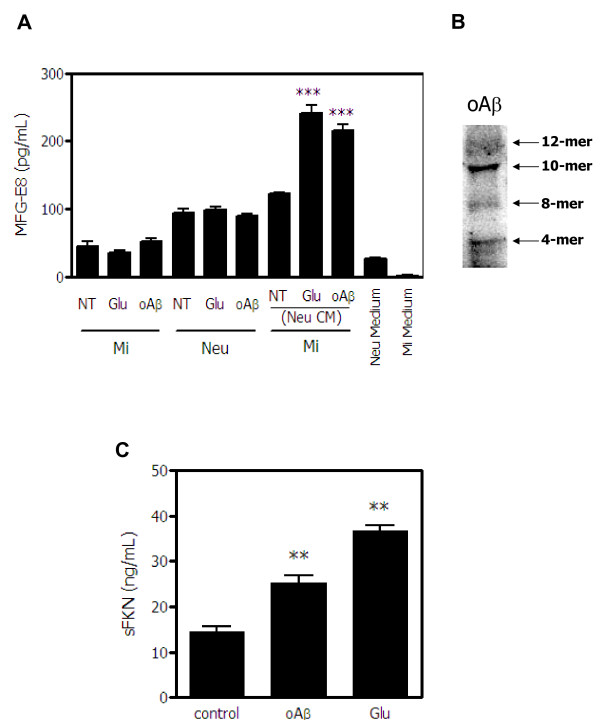
**Induction of MFG-E8 by soluble factors released from degenerated neurons. (A)** The levels of MFG-E8 secreted from microglia (Mi) and cortical neurons (Neu) treated with 20 μM glutamate (Glu) or 5 μM oligomeric amyloid β (oAβ) were measured by ELISA. In addition, MFG-E8 released from microglia exposed to neuronal conditioned medium (Neu CM) that had been treated with Glu or oAβ was also measured. Results are presented as the means with S.E.M. (n = 3). Glu- or oAβ-treated neuronal conditioned medium significantly induced MFG-E8 expression from microglia compared with the untreated control samples. ***: *P* <0.001 (one-way ANOVA with Dunnett’s *post-hoc* test). **(B)** The Western blot data of oAβ used in the present study. The blot was incubated in mouse anti-Aβ monoclonal antibody (6E10) (1:1,000, Chemicon). **(C)** The levels of the soluble secreted form of fractalkine (sFKN) released from cortical neurons treated with 20 μM glutamate (Glu) or 5 μM oligomeric amyloid β (oAβ) were measured. The results are presented as the means with S.E.M. (n = 3). Glu and oAβ treatment significantly induced sFKN release from neurons compared to the untreated control samples. **: *P* <0.01 (one-way ANOVA with Dunnett’s *post-hoc* test).

We first examined whether microglia produce MFG-E8 in response to soluble factors released from degenerated neurons. The direct glutamate or oAβ treatment did not induce MFG-E8 secretion from either microglia or neurons. The MFG-E8 levels in the culture medium for neurons or microglia were negligible (Figure 1A). However, the levels of MFG-E8 secreted from microglia significantly increased when the cells were treated with conditioned medium from neurons that had been exposed to glutamate or oAβ (Figure 1A). The Western blot analysis for oAβ used in the experiments was shown in Figure 1B. Neurons released significant amounts of soluble fractalkine (sFKN) when exposed to oAβ as well as glutamate (Figure 1C). In a previous study, we showed that sFKN is secreted from glutamate-damaged neurons and induces MFG-E8 expression in microglia [3], suggesting that sFKN may play a role as a mediator to induce MFG-E8 release from microglia.

### MFG-E8 directly induces microglial neuroprotective effects

**Figure 2:**
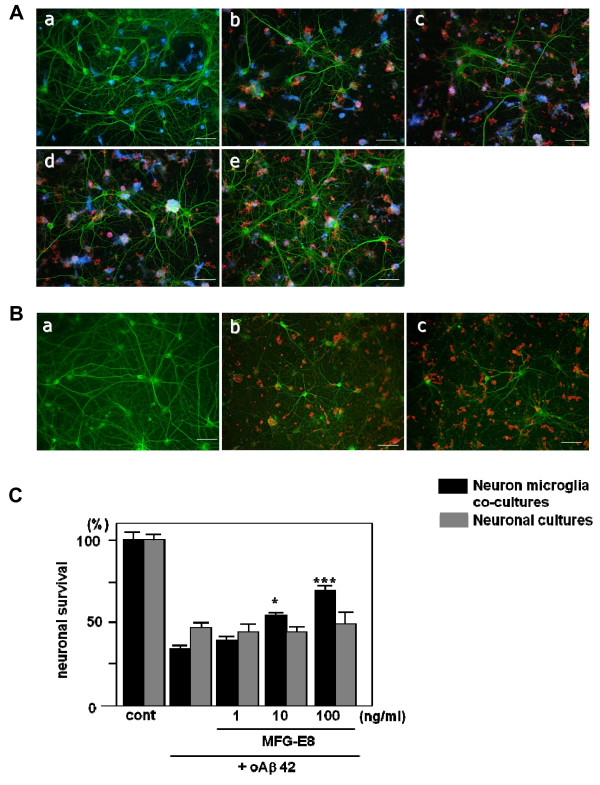
**MFG-E8 exerts neuroprotective effects in the presence of microglia.** The effect of MFG-E8 treatment against oAβ toxicity in both neuron − microglia co-cultures **(A)** and neuronal cultures **(B)**. Neurons were stained with an anti-MAP-2 antibody (green), microglia were stained with a Cy5-conjugated anti-CD11b antibody (*blue*), and oAβ was stained with an anti-4 G8 antibody (*red*). Scale bar = 50 μm. Untreated neuronal cultures and neuron − microglia cocultures (1:2 neurons to microglia) (cont; *a*). Five micromolar of oAβ induced neuronal loss in both neuronal and neuron–microglia cocultures (*b*). In the presence of microglia, MFG-E8 treatment induced neuroprotective effects in a dose-dependent manner (doses were 1 ng/mL (*c*), 10 ng/mL (*d*), 100 ng/mL (*e*) in (A)). On the other hand, MFG-E8 did not show any effects on oAβ-induced neurotoxicity, even at the highest dose (100 ng/mL; *c* in (B)). **(C)** Neuronal survival was estimated as the percentage of intact neurons in the treated sample relative to the untreated sample. The results are presented as the means with S.E.M. (n = 3), in which 10 randomly selected fields were analyzed. Significant differences compared to samples only treated with oAβ are noted. *: *P* <0.05, ***: *P* <0.001, (one-way ANOVA with Dunnett’s *post-hoc* test).

We then examined the direct effects of MFG-E8 on neuronal survival. There has been little evidence indicating that MFG-E8 exerts neuroprotective effects, aside from our previous report in which neutralizing MFG-E8 markedly attenuated sFKN-induced neuroprotection [3]. Therefore, we first determined whether MFG-E8 has direct neuroprotective effects against oAβ toxicity in neuron–microglia cocultures (Figure 2A*a − e*) or in neuronal cultures (Figure 2B*a − c*). Treating neurons with 5 μM oAβ for 24 h induced obvious cell death in both neuron − microglia cocultures (Figure 2A*b,* C) and neuron cultures (Figure 2B*b,* C). MFG-E8 significantly inhibited oAβ-induced cell death in a dose-dependent manner in neuron − microglia cocultures (Figure 2A*c* − *e,* C), but not in neuron cultures (Figure 2B*c,* C).

### MFG-E8 increases phagocytosis of oAβ through a CD47-mediated signaling pathway in microglia

**Figure 3:**
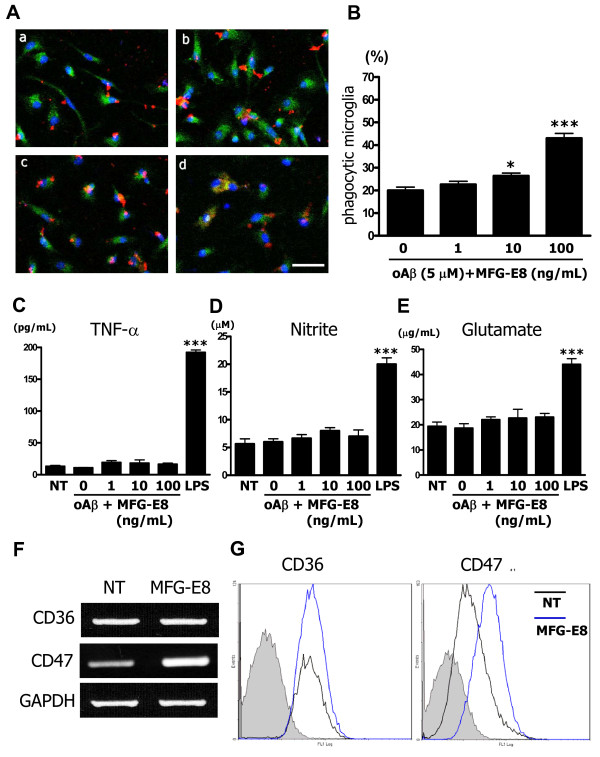
**MFG-E8 induces microglial phagocytosis of oAβ. (A)** Microglia were pre-treated with 1 ng/mL (*b*), 10 ng/mL (*c*) or 100 ng/mL (*d*) MFG-E8 for 1 h, and then 5 μM oAβ was added to the culture for 6 h. Primary antibodies recognizing Rab7 (*green*), oAβ (*red*) and nuclei (*blue*) were used (*a* − *d*). A few oAβ-incorporated microglia were detected in the absence of MFG-E8 (*a*), while a sufficient number of microglia that had engulfed oAβ were detected in the presence of the higher MFG-E8 dose (*d*) under confocal laser scanning microscope. Scale bar = 50 μm. **(B)** Quantification of the phagocytosis index, which is defined as the percentage of total microglial-Rab7 staining (*green*) that overlaps with oAβ staining (*red*). The results are presented as the means with S.E.M. (n = 3), in which 10 randomly selected fields were analyzed. Significant differences compared with samples that were only treated with oAβ (oAβ) are noted. *: *P* <0.05 (one-way ANOVA with Dunnett’s *post-hoc* test). The measurement of TNF-α **(C)**, nitrite **(D)**, and glutamate **(E)** produced by microglia treated with MFG-E8 plus oAβ was performed. After 24 h treatment with 1 ng/mL, 10 ng/mL or 100 ng/mL MFG-E8 with 5 μM oAβ, the supernatants of microglial cultures were analyzed. 100 ng/mL LPS treatment for 24 h was used as a positive control. The results are presented as the means with S.E.M. (n = 5). Significant differences compared to untreated microglia (NT) are noted. ***: *P* <0.001, (one-way ANOVA with Dunnett’s *post-hoc* test). **(F)** mRNA expression levels of oAβ phagocytosis-related cellular membrane surface antigens, CD36 and CD47, in microglia treated with 100 ng/mL MFG-E8 for 24 h was assessed using RT-PCR. GAPDH expression was used as a control. (D) CD36 and CD47 protein levels were assessed by FACS analysis. Microglia were treated with 100 ng/mL MFG-E8 for 72 h. The gray-filled curve represents the isotype-matched control. The black and blue lines indicate untreated (NT) and MFG-E8-treated samples, respectively.

To explore the detailed mechanisms by which MFG-E8 exerts neuroprotection in the presence of microglia, we investigated whether MFG-E8 increased microglial phagocytosis of Aβ under confocal laser scanning microscope. Non-treated microglia minimally engulfed Aβ, as determined by the co-localization of the phagosome maturation marker Rab-7 and Aβ (Figure 3A*a*). However, MFG-E8 increased microglial phagocytosis of Aβ in a dose-dependent manner (Figure 3A*b* − *d*). The ratio of phagocytic to total cells is shown graphically (Figure 3B) and treating with 10 ng/mL and 100 ng/mL MFG-E8 significantly increased this ratio. The neurotoxic molecules, such as TNF-α, NO, and glutamate, were not induced by MFG-E8- induced microglial phagocytosis of Aβ (Figure 3C-E).

We next examined the effects of MFG-E8 on the expression of principal phagocytosis-related surface receptors in microglia. The expression of the classical microglial phagocytosis receptors CD14 and TLR4, which are LPS receptors, was unchanged with MFG-E8 treatment, and TLR6 and TLR9 expression was also unchanged (data not shown). In AD brains, microglia reportedly interact with amyloid plaque by forming functional receptors including α_6_β_1_ integrin, CD36 and CD47, which act concertedly to engulf Aβ [19,29]. Thus, we next examined the effect of MFG-E8 on CD36 and CD47 expression in microglia. Treating microglia with 100 ng/mL of MFG-E8 markedly induced CD47 mRNA expression but not CD36 expression (Figure 3F). FACS analysis for CD36 and CD47 protein levels yielded similar results; treating microglia with MFG-E8 for 72 h increased microglial CD47 expression, but not CD36 expression (Figure 3G).

**Figure 4:**
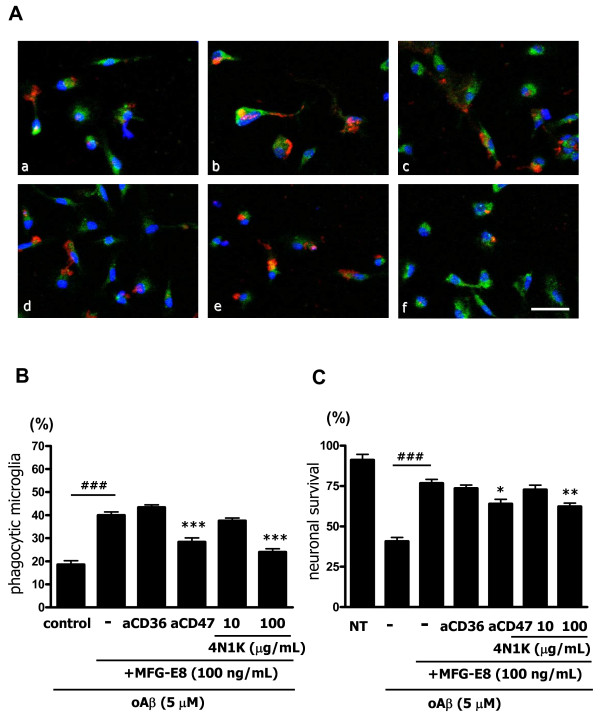
**MFG-E8 enhances microglial phagocytosis of oAβ through CD47. (A)** Microglia were treated with 1 μg/mL of isotype-matched control IgG (*a, b*), 100 ng/mL MFG-E8 (*b − f*), 1 μg/mL of anti-CD36 neutralizing antibody (*c*), 1 μg/mL of anti-CD47 neutralizing antibody (*d*), the peptide antagonist 4N1K 10 μg/mL (*e*) or 100 μg/mL (*f*) for 1 h, and then 5 μM oAβ was added to the culture for 24 h. Primary antibodies against Rab7 (*green*), oAβ (*red*) and nuclei (*blue*) were used (*a* − *f*). Scale bar = 50 μm. **(B)** Quantification of the phagocytosis index. Results show the means with S.E.M. (n = 3), in which 10 randomly selected fields were analyzed. Significant differences compared with the isotype-matched control samples (#) or samples treated only with MFG-E8 (*) are noted. ***: *P* <0.001; ###: *P* <0.001 (one-way ANOVA with Tukey’s *post-hoc* test). **(C)** The effects of blockade of CD36 and CD47 in MFG-E8-treated microglia on neuronal survival. Neuron − microglia co-cultures were pre-treated with MFG-E8 for 1 h in the presence of anti-CD36 neutralizing antibody, anti-CD47 neutralizing antibody, and 10 μg/mL or 100 μg/mL 4N1K. Then, the cultures were treated with oAβ for 24 h. The neuronal survival rate was estimated. The results are presented as the means with S.E.M. (n = 5), in which 10 randomly selected fields were analyzed. Significant differences compared with the isotype-matched control samples (#) or samples treated only with MFG-E8 (*) are noted. *: *P* <0.05; ###: *P* <0.001 (one-way ANOVA with Tukey’s *post-hoc* test).

Then, we examined the involvement of these cellular surface antigens in Aβ phagocytosis. MFG-E8 (100 ng/mL) increased phagocytosis almost two-fold relative to the unstimulated control (Figure 4A*b,* B). Although blocking CD36 with a specific antibody did not have an effect (Figure 4A*c,* B), blocking CD47 with a specific antibody completely suppressed MFG-E8 − induced phagocytosis (Figure 4A*d,* B). High concentrations of 4N1K, a CD47 blocking peptide [30], similarly inhibited phagocytosis (Figure 4A*ef*, B). These data suggest that Aβ was recognized and phagocytosed by microglia through a CD47-mediated pathway. Moreover, we examined whether blockade of CD47 affects neuronal survival by MFG-E8-treated microglia. We confirmed that blockade of CD47 with anti-CD47 antibody or 4N1K decreased neuronal survival (Figure 4C).

### MFG-E8 induces neuroprotective effects in microglia through the Nrf2-HO-1 pathway

**Figure 5:**
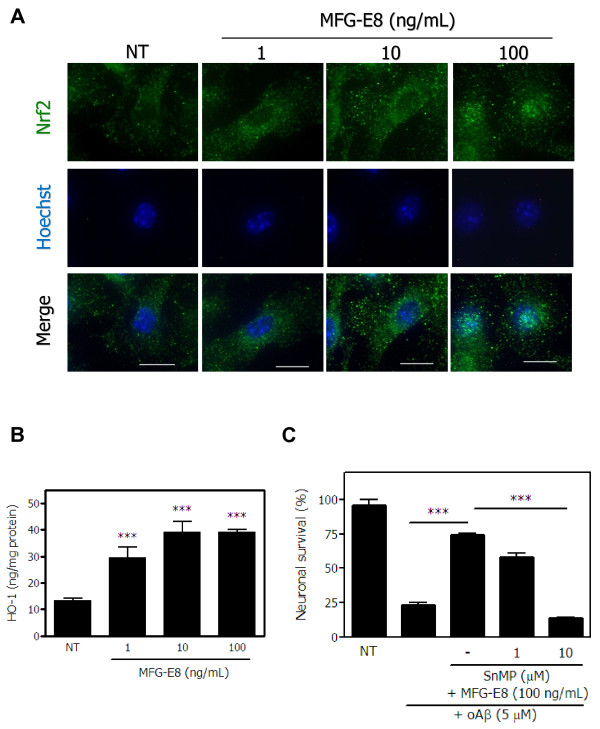
**MFG-E8 exerts neuroprotective effects by activating the Nrf-2-HO-1 pathway in microglia. (A)** Immunofluorescence images of Nrf2 (*green*) and nuclei (Hoechst; *blue*) in microglia treated with the indicated concentrations of MFG-E8. Scale bar = 10 μm. **(B)** After microglia were treated with the indicated concentrations of MFG-E8 for 24 h, HO-1 protein expression levels were measured by ELISA. Significant differences compared with untreated samples (*) are noted. ***: *P* <0.001 (one-way ANOVA with Dunnett’s *post-hoc* test). **(C)** Neuron − microglia co-cultures were pre-treated with MFG-E8 for 3 h in the presence of the HO-1 inhibitor SnMP. Then, the cultures were treated with oAβ for 24 h. The neuronal survival rate in the presence of MFG-E8 and SnMP was estimated. The results are presented as the means with S.E.M. (n = 3), in which 10 randomly selected fields were analyzed. Significant differences compared with oAβ plus MFG-E8 treated samples are noted. ***: *P* <0.001 (one-way ANOVA with Dunnett’s *post-hoc* test).

We previously showed that neuroprotection by sFKN is initiated by the nuclear translocation of the transcription factor Nrf2 and subsequent HO-1 production [3]. Thus, we examined whether MFG-E8 induces a change in the subcellular localization of Nrf2. MFG-E8 markedly enhanced the nuclear translocation of Nrf2 in a dose-dependent manner, as determined by immunostaining (Figure 5A). In addition, MFG-E8 also dose-dependently increased HO-1 production (Figure 5B). However, the expression of another Nrf2-induced phase-II enzyme, NAD(P)H quinine oxidoreductase-1 (NQO-1), was not changed by MFG-E8 (data not shown). Finally, we examined the role of HO-1 in MFG-E8 − induced neuroprotection against Aβ toxicity. The neuroprotective effects of MFG-E8 were abolished by treating with 10 μM SnMP, an HO-1 inhibitor, although a lower concentration of SnMP did not exert these effects (Figure 5C). We also examined the expression of neuroprotectants other than HO-1 and NQO-1. MFG-E8 did not affect the mRNA levels of BDNF, GDNF, NGF, TGF-β or IL-10 (data not shown ). Therefore, Nrf2-induced HO-1 production by microglia may contribute to the neuroprotective effects of MFG-E8.

## Discussion

In this study, we showed for the first time that MFG-E8 has neuroprotective effects against oAβ toxicity by enhancing microglial phagocytic activity and exerting anti-oxidant effects. MFG-E8 was initially described as a mouse mammary epithelial cell surface protein but was more recently identified as a glycoprotein secreted from macrophages in germinal centers of the spleen and lymph nodes [1]. Phagocytosis is thought to be induced by “eat-me” signals, such as the nucleotide GDP and PS. MFG-E8 is a PS receptor and promotes apoptotic cell clearance [31]. In the present study, it is clear that MFG-E8 also plays a role in the engulfment of Aβ by microglia. It has been shown that MFG-E8 enhances phagocytosis of UV-treated apoptotic neurons by microglia [32], and that a MFG-E8/lactadherin deficiency markedly reduces Aβ phagocytosis by macrophages [4]. We also revealed that microglial phagocytosis of Aβ is mediated through CD47 expressed on microglia. Other microglial phagocytosis-related receptors, such as CD14, and components of the senile plaque-interacted receptor, such as CD36, were not affected by MFG-E8. Although both CD14 and CD36 have been associated with MFG-E8 [33] or microglial Aβ phagocytosis [16], they were not induced by MFG-E8 treatment in this study. Blocking CD47 with a neutralizing antibody or the 4N1K peptide significantly reduced Aβ phagocytosis. The CD47 ligand is a signal regulatory protein-α (SIRPα) that is expressed on the cellular membrane of neurons as well as glial cells [17]. SIRPα is also reportedly a negative regulator of the CD47-mediated signaling pathway [17,18]. We previously showed that MFG-E8 expression is induced by sFKN released from degenerated neurons [3]. sFKN increased HO-1 production via the JNK MAPK-Nrf2 pathway in microglia and exerted neuroprotection against excitotoxicity. In this study, MFG-E8 increased the nuclear translocation of Nrf2 and enhanced HO-1 production in microglia. Since the expression of another Nrf2-induced anti-oxidative enzyme, NQO-1, was not increased by MFG-E8, HO-1 might mediate MFG-E8 − induced neuroprotection against Aβ toxicity. Accordingly, MFG-E8 may be downstream of sFKN in microglia. Aβ-induced oxidative stress is mediated through ROS production by glial cells and also by neurons [20,21,34-36]. ROS may lead to neuronal degeneration in AD. These results delineate a novel function for MFG-E8 in neuroprotection by microglia.

oAβ is toxic to neuronal cells in the present study. It has been reported that Aβ also enhances microglial activation to produce pro-inflammatory molecules [37-40]. oAβ induces microglia to have a pro-inflammatory character [37], and increases microglial TNF-α secretion through a tyrosine kinase dependent pathway [39]. NO-mediated microglial neurotoxicity by oAβ is reported [40]. oAβ disturbs microglial phagocytosis and clearance of fibrillar Aβ [15].

However, oAβ did not induce inflammatory molecules, such as TNF-α, NO and glutamate in microglia in the present and previous studies [24]. The synthetic oAβ used in the present study contains oligomers of different sizes and shapes, so that microglia may respond to this heterogeneous oAβ in various ways. Furthermore, synthetic oAβ is less potent than oAβ isolated from the transfected cell cultures.

Since MFG-E8 is a ligand for α_v_β_3_ and α_v_β_5_ integrins, MFG-E8 presumably interacts with microglia by binding to integrins [1,41]. MFG-E8 forms a cellular bridge between the integrin on phagocytes and PS on target cells. MFG-E8 induces CD47 expression in microglia. CD47 directly interacts with Aβ to form a complex that induces microglial engulfment [29], after which MFG-E8 increases Aβ clearance via CD47. RAGE is a surface molecule that directly interacts with Aβ and activates NFκB-mediated signaling pathways in microglia [42,43]. Recent reports showed that RAGE also directly interacts with PS and enhances macrophage phagocytosis [44]. RAGE simultaneously enhances the inflammatory status. On the other hand, MFG-E8 enhances microglial phagocytosis without inducing inflammation.

**Figure 6:**
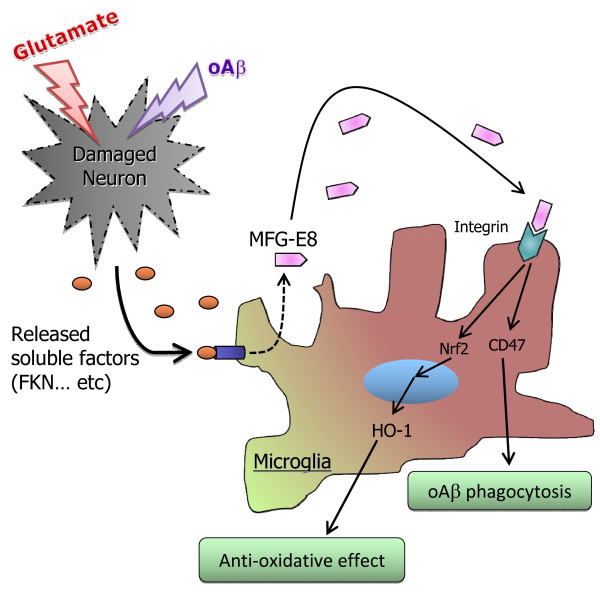
**Model of the neuroprotective role of MFG-E8 against Aβ toxicity.** MFG-E8 is secreted from microglia in response to soluble factors (for example, FKN) produced by degenerated neurons. MFG-E8 enhances Aβ phagocytosis by microglia through CD47 expression. MFG-E8 also directly increases the nuclear translocation of the transcriptional factor Nrf2, which induces expression of the antioxidant enzyme HO-1. Therefore, MFG-E8 is a potent downstream molecule of sFKN and is an intrinsic neuroprotectant for damaged yet surviving neurons.

Our data are schematically summarized in Figure 6. Glutamate and oAβ damage neurons, which then release soluble factors, including FKN, as “help-me” signals. In response to these factors, microglia release MFG-E8. MFG-E8 directly increases Aβ phagocytosis by microglia via CD47 expression and also increases HO-1 expression in microglia via nuclear translocation of the transcription factor Nrf2. Each of these processes contributes to microglial neuroprotection. Therefore, MFG-E8 or agents that increase its expression may be potential therapeutic targets for neurodegenerative diseases, including AD.

## Conclusions

Here we show that MFG-E8 is released from microglia in response to soluble factors (for example, FKN) secreted from degenerated neurons. MFG-E8 induces microglial neuroprotective activity against oAβ toxicity. This neuroprotection is mediated through enhanced microglial phagocytic activity via the CD47 pathway and progression of the Nrf2-HO-1 pathway. These data indicate that MFG-E8 may have novel roles as a neuroprotectant that is produced from microglia during neurodegenerative conditions.

## Abbreviations

AD, Alzheimer’s disease; CNS, Central nervous system; FKN, Fractalkine; GAPDH, Glyceraldehydes-3-phosphate dehydrogenase; HO-1, Hemeoxygenase-1; LPS, Lipopolysaccharide; MFG-E8, Milk fat globule-EGF factor 8; NO, Nitric oxide; NQO-1, NAD(P)H quinine oxidoreductase-1; Nrf2, Nuclear factor E(2)-related factor 2; oAβ, Oligomeric amyloid β; PS, Phosphatidylserine; RAGE, Receptor for advanced glycation end products; ROS, Reactive oxygen species; TLRs, Toll-like receptors; TNF-α, Tumor necrosis factor-α.

## Competing interests

The authors declare that they have no competing interests.

## Authors’ contributions

EL performed the RT-PCR experiments and helped draft the manuscript. MN conducted the ELISAs, microglial phagocytosis assay and FACS analysis, and drafted the manuscript. YD, BP, JK and HT performed the cell culture and were involved in the conception of the study. TM carried out the immunocytochemistry. He was also involved in the conception and design of the study, and helped draft the manuscript. AS was also involved in the conception and design of the study, as well as in the preparation of the manuscript. All authors read and approved the final manuscript.
